# AF-RT-DETR: Adaptive cross-scale feature interaction for real-time plant disease detection in complex field environments

**DOI:** 10.3389/fpls.2026.1788655

**Published:** 2026-04-10

**Authors:** Ming Liu, Jiangrong Liu, Ziqi Mao, Shuzhe Cheng, Xinyang Li, Shiyu Yan

**Affiliations:** 1 School of Mathematics and Computer Science, Wuhan Polytechnic University, Wuhan, China; 2 School of Computer and Artificial Intelligence, Wuhan Textile University, Wuhan, China; 3 China Railway 11th Bureau Group Xi’an Construction Co., Ltd., Xi’an, China

**Keywords:** disease detection, feature extraction, feature fusion, object detection, RT-DETRv2

## Abstract

**Introduction:**

Accurate plant disease identification is of great importance for ensuring agricultural productivity and food security. However, complex illumination variations, leaf occlusion, and diverse disease spot scales throughout plant growth stages significantly increase the difficulty of real-time detection, leading to limited accuracy and robustness in existing approaches.

**Methods:**

To address these challenges, we propose an improved RT-DETRv2-based plant disease detection model, termed AF-RT-DETR. A Bidirectional Cross Gate (BCG) module is introduced in the feature extraction stage to reduce channel redundancy and enhance discriminative feature representation through multi-level feature interactions. The original RepVGG structure is replaced with a Dynamic Channel Shift (DCS) module, effectively enlarging the receptive field and strengthening contextual feature fusion without additional computational overhead. Additionally, an improved Scale-aware Multi-level Loss (SML) emphasizes low-quality feature maps to improve detector robustness.

**Results:**

The model achieves mAP50 and mAP50:95 of 93.6% and 67.2% on the Plant-Disease dataset, surpassing the baseline by 5.1% and 4.5%. Furthermore, the model was evaluated on multiple crops and growth stages under diverse field conditions, demonstrating robust performance and adaptability.

**Discussion:**

These results indicate that AF-RT-DETR effectively enables real-time plant disease detection in complex field environments.

## 1 Introduction

Plant diseases are among the most critical factors affecting agricultural productivity and global food security, as they directly influence crop yield, quality, and the stability of agri-food supply systems (Vyska et al., 2016; Chai et al., 2022) With the intensification of climate change and the expansion of large-scale agricultural practices, crops are increasingly exposed to pathogens such as fungi, bacteria, viruses, and pests during their growth cycle, leading to symptoms including leaf wilting, fruit decay, and stem rot (Raza and Bebber, 2022). If plant diseases are not detected and controlled at an early stage, they may result in severe yield losses or even complete crop failure, posing substantial risks to farmers’ income and the resilience of global food supply chains (Kalantari Khalil Abad et al., 2025). Therefore, timely and accurate detection of plant diseases under complex field conditions is essential for sustainable agricultural development.

Traditional plant disease identification methods mainly rely on manual field inspection and expert experience. Although effective in small-scale scenarios, these approaches suffer from limited coverage, low efficiency, and strong dependence on subjective judgment and individual expertise (Sun et al., 2023; Chen et al., 2024). To address these limitations, automated detection techniques based on imaging and sensing technologies, such as visible light imaging, multispectral imaging, and hyperspectral imaging, have been extensively explored (Singh et al., 2020; Christopoulou et al., 2024). Among them, multispectral and hyperspectral imaging can capture spectral responses of plants across multiple wavelength bands, enabling early detection of disease symptoms that are difficult to observe visually (Xuan et al., 2022). However, the high cost of imaging equipment, complex data processing pipelines, and sensitivity to illumination and environmental variations hinder their widespread adoption in large-scale field applications.

With the rapid advancement of deep learning, object detection methods based on CNNs and Transformers have become the dominant paradigm for intelligent plant disease detection. Two-stage detectors such as Faster R-CNN (Ren et al., 2017) generate candidate regions via region proposal networks and have demonstrated high detection accuracy in various crop disease detection tasks, especially when combined with attention mechanisms and multi-scale feature fusion strategies (Liu et al., 2021).Recent studies have further explored attention-enhanced deep architectures for agricultural image analysis. For example, Khan et al. proposed a Dense-Inception architecture with attention modules for plant disease classification, demonstrating the effectiveness of attention mechanisms in capturing discriminative disease patterns in plant images (Khan et al., 2025). Nevertheless, their relatively high computational complexity and slow inference speed limit their applicability in real-time field scenarios.

Single-stage detectors represented by the YOLO family reformulate object detection as an end-to-end regression problem, achieving a favorable balance between detection accuracy and inference speed. Improved YOLOv5-based models have been successfully applied to vegetable and leaf disease detection, maintaining robust performance under complex illumination and background conditions (Li et al., 2022). Subsequent versions, including YOLOv8 (Ultralytics, [[NoYear]]), YOLOv9 (Wang et al., 2024), and YOLOv10 (Wang et al., 2024), further enhanced feature representation and gradient propagation through architectural and training strategy improvements. Recent benchmark studies have also systematically evaluated different YOLO variants in agricultural environments, demonstrating their effectiveness in tasks such as crop growth monitoring and weed detection in field imagery (Raza et al., 2025). Despite these advances, YOLO-based detectors generally rely on non-maximum suppression (NMS) for post-processing, making their performance sensitive to threshold selection and less stable in dense and highly occluded field environments.

To overcome the limitations of anchor-based designs and heuristic post-processing, Carion et al (Carion et al., 2020). introduced DETR, an end-to-end object detection framework based on the Transformer architecture (Vaswani et al., 2017), which leverages self-attention mechanisms to model global contextual information and directly outputs predictions via bipartite matching. DETR and its variants (Li and Shi, 2024) have been applied to plant disease and crop-related detection tasks, demonstrating strong global modeling capability in scenarios with overlapping leaves and complex backgrounds. Beyond object detection, attention-enhanced encoder–decoder architectures have also been explored in related agricultural vision tasks, such as crop–weed segmentation in UAV imagery, highlighting the potential of attention mechanisms for modeling complex field scenes (Khan et al., 2023). However, the original DETR suffers from slow training convergence and high dependence on large-scale annotated datasets.

To address these issues, several improved DETR-based models have been proposed. Deformable DETR (Zhu et al., 2021) reduces computational complexity through deformable attention mechanisms, making it more suitable for high-resolution field images. Conditional DETR (Meng et al., 2021) accelerates convergence by introducing conditional query embeddings, while DN-DETR (Li et al., 2022) and DINO (Zhang et al., 2023) stabilize bipartite matching through denoising training strategies. Moreover, DAB-DETR (Liu et al., 2022) and Lite DETR (Li et al., 2023) focus on query design and structural optimization to achieve a better trade-off between detection accuracy and inference efficiency. Despite these improvements, Transformer-based detectors still face challenges in computational efficiency and small-scale disease spot modeling under complex field conditions.

In this context, we propose a new model, AF-RT-DETR, based on RT-DETRv2, to enhance real-time plant disease detection under complex field conditions. The main contributions of our work are as follows:

1. To enhance multi-scale feature interaction and suppress redundant information, we incorporate a bidirectional cross-gating mechanism into the feature extraction stage.
2. To strengthen contextual feature aggregation and improve detection accuracy without increasing the overall parameter scale, we introduce a dynamic channel selection strategy.
3. To guide query-object matching more effectively and improve training stability for low-quality matches, we adopt an improved loss function addressing limitations of existing losses such as Focal Loss, Varifocal Loss, and Matchability-Aware Loss.

## 2 Materials and methods

### 2.1 Baseline model

RT-DETR is a real-time end-to-end object detector proposed by the Baidu research team, aiming to bring the advantages of the DETR framework into practical real-time applications. Its core design introduces an efficient Hybrid Encoder that decouples intra-scale feature interaction from cross-scale feature fusion, enabling effective feature representation with reduced computational overhead. In addition, RT-DETR adopts an uncertainty-minimization-based query selection strategy to provide high-quality initial queries for the decoder, achieving a favorable trade-off between detection accuracy and inference speed and serving as an important reference for real-time Transformer-based detectors.

**Figure 1:**
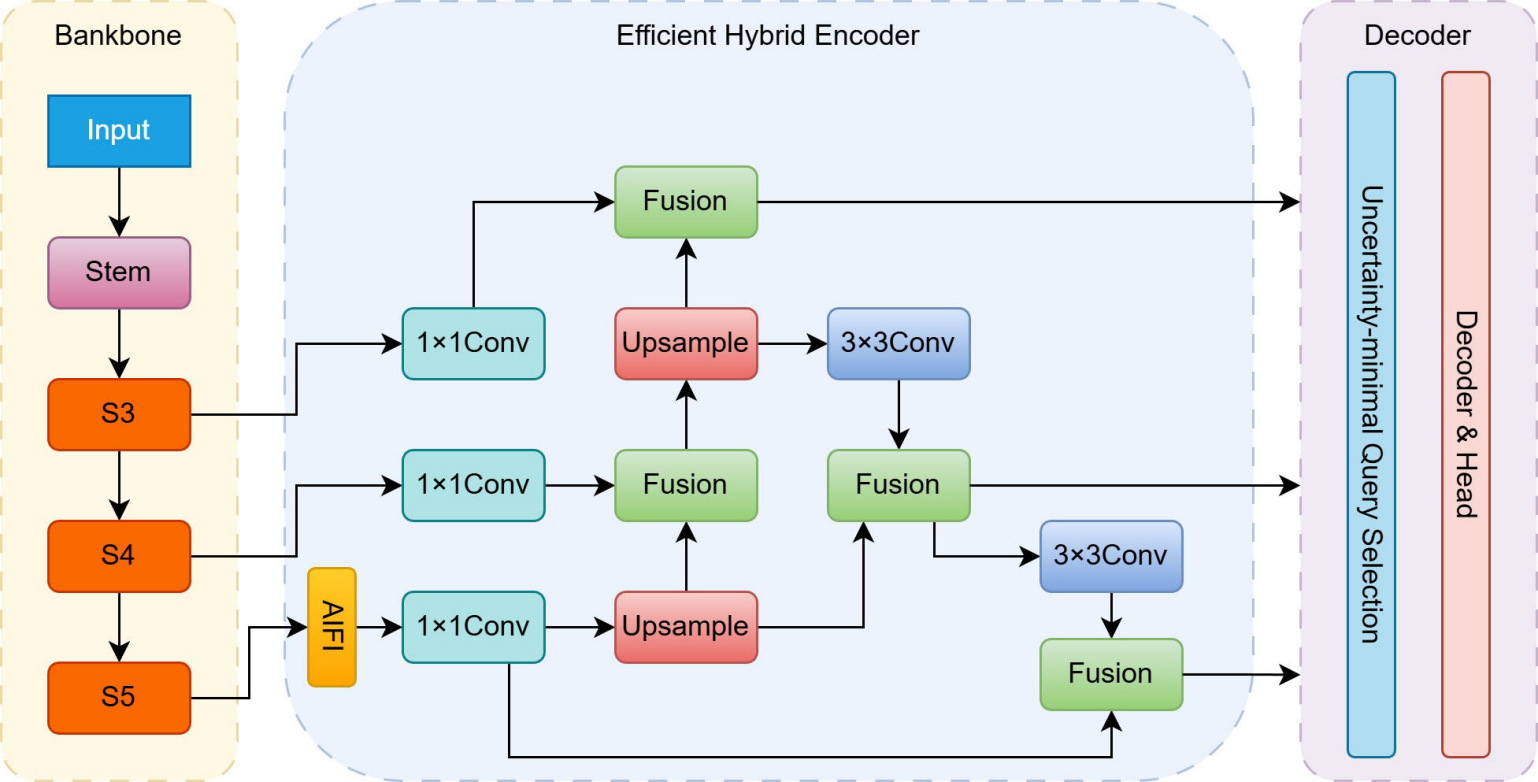
RT-DETRv2 module structure.

Building upon RT-DETR, RT-DETRv2 (**Figure 1**) further optimizes the model architecture and training strategy to enhance flexibility and deployment practicality while preserving the overall framework. Specifically, RT-DETRv2 assigns adaptive numbers of sampling points to different feature scales in the decoder, enabling more precise multi-scale feature modeling. Moreover, an optional discrete sampling operator is introduced to replace the original grid_sample operation, effectively removing deployment constraints associated with specialized operators and facilitating efficient inference across diverse hardware platforms. In addition, RT-DETRv2 incorporates dynamic data augmentation and scale-adaptive training strategies to further exploit the model’s potential across varying input resolutions.

### 2.2 Proposed model

RT-DETRv2 adopts a relatively straightforward strategy for multi-scale feature modeling. Specifically, feature maps from different backbone stages are first aligned in spatial resolution via upsampling or downsampling and then fused through direct concatenation. This design is computationally efficient and well-suited for real-time detection tasks, particularly for deployment on edge devices. However, such scale-aligned concatenation-based fusion may introduce channel redundancy and limit the model’s ability to adaptively exploit multi-level semantic information, especially in complex natural environments. In field-based plant disease detection scenarios characterized by variable illumination, cluttered backgrounds, and large scale variations of lesions, these limitations tend to become more pronounced.

To address this issue, this study introduces a BCG module at the feature extraction stage. By enabling bidirectional information interaction and selective enhancement across feature maps from different stages, the BCG module facilitates more effective modeling of spatial and channel-wise dependencies. This design helps suppress redundant information while improving the discriminative capability of multi-scale feature representations.

In addition, RT-DETRv2 employs a structural re-parameterization strategy during feature fusion to accelerate inference. Although the multi-branch architecture benefits optimization stability during training, it is ultimately equivalent to a single convolutional operator at inference time, resulting in feature representations that remain constrained by a local receptive field and limited capacity for capturing long-range contextual information. Moreover, the re-parameterization process tends to obscure the relative importance of different channels, which may hinder fine-grained feature fusion.

To overcome these limitations, we propose a DCS module, which introduces adaptive channel-wise rearrangement and cross-channel interaction mechanisms. Without increasing computational complexity, DCS effectively enlarges the receptive field and enhances contextual feature aggregation. Together, the BCG and DCS modules constitute the core components of the proposed improved RT-DETRv2 framework. The detailed architectures and implementation of these modules are presented in Sections 2.3 and 2.4, respectively.

### 2.3 Bidirectional cross gate

**Figure 2:**
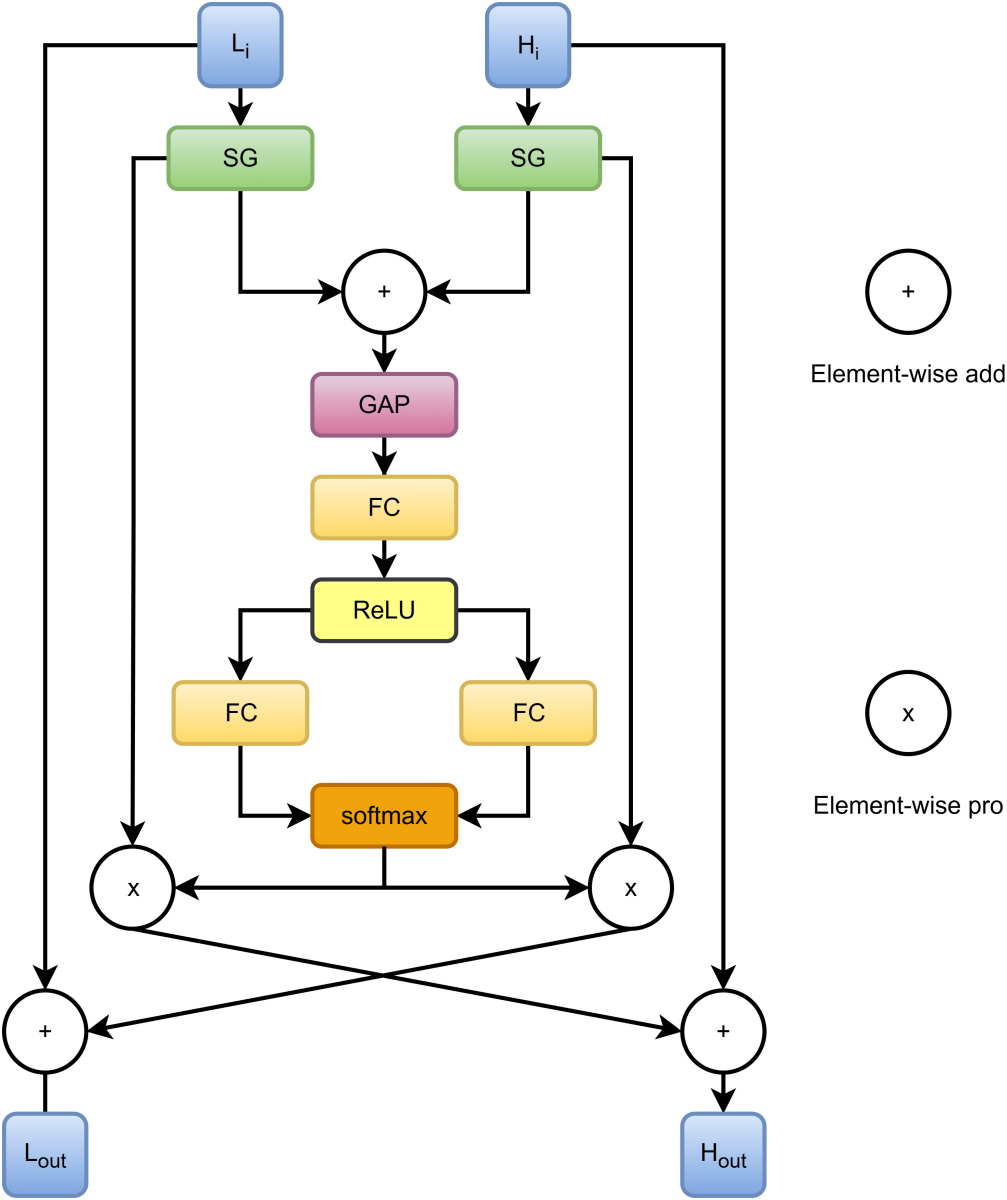
Bidirectional cross gate module structure.

To enable efficient multi-scale feature fusion, we propose the BCG module, whose architecture is illustrated in **Figure 2**. The module realizes fine-grained bidirectional interaction between high-level and low-level features through a gate-controlled residual injection mechanism. Let the input high-level feature be $H\;\in \;R^{B\times C\times H\times W}$ and the low-level feature be $L\;\in \;R^{B\times C\times H\times W}$,where B, C, H, W denote the batch size, channel number, height, and width, respectively. The BCG module outputs the enhanced features $H_{out}$ and $L_{out}$, which preserve the same spatial and channel dimensions as the inputs. The forward process of the module consists of three main steps.

Spatial Information Refinement.In this stage, a spatial gate unit SG(·) is applied to reweight the input features, generating spatial attention maps that emphasize informative regions while suppressing irrelevant responses. The refinement process can be formulated as (Equations 1, 2):

(1) $$\tilde{H}=SG(H)⊗H$$

(2) $$\tilde{L}=SG(L)⊗L$$

Where ⨂ denotes element-wise multiplication.

Channel Weight Generation.To control the intensity of bidirectional feature injection, channel-wise gate weights are further computed. First, the refined features are fused by element-wise addition (Equation 3):

(3) $$U=\tilde{H}+\tilde{L}$$

Then, global average pooling GAP (·) is employed to squeeze the spatial dimensions and obtain a channel-level statistical descriptor (Equation 4):

(4) S=GAP(U)

Based on S, a bottleneck structure is introduced to generate channel weights. Specifically, a linear projection is first used to reduce the channel dimension, followed by a ReLU activation (Equation 5):

(5) $$Z=ReLU(W_{1}S)$$

Subsequently, two independent linear projections are adopted to generate the raw weight vectors corresponding to the high-level and low-level branches (Equations 6, 7):

(6) $$a_{h}=W_{1}Z$$

(7) $$a_{l}=W_{2}Z$$

where ${\text{W}}_{\text{i}}$ denotes learnable linear projection parameters.

Finally, the raw weights are normalized along the branch dimension using the Softmax function (Equation 8):

(8) $$\left[w_{h},w_{l}\right]=Softmax(\left[a_{h},a_{l}\right]$$

yielding normalized channel gate weights.

Bidirectional Gated Injection.In the final stage, bidirectional feature injection is performed in a residual manner. A learnable global injection strength coefficient α is introduced and initialized to zero at the beginning of training to ensure stable optimization. The output features are computed as:

(9) $$H_{out}=H+\text{α}\cdot \left(w_{l}⊙\tilde{L}\right)$$

(10) $$L_{out}=L+\text{α}\cdot \left(w_{h}⊙\tilde{H}\sim \right)$$

Where ⊙denotes element-wise multiplication. Equation (9) indicates that low-level features are injected into high-level features under the control of channel gates to supplement fine-grained details, while Equation (10) injects high-level semantic information into low-level features to enhance semantic consistency.

### 2.4 Dynamic channel shift

As discussed in Section 3.1, the feature modeling capability of RT-DETRv2 during feature fusion is partly constrained by the limited receptive field of convolution kernels. To address this issue, several studies have explored receptive field expansion. shiftViT (Wang et al., 2022) replaces self-attention with parameter-free shift blocks and maintains competitive performance in lightweight models; however, its limited receptive field restricts effective long-range dependency modeling in complex scenes. RepLKNet (Ding et al., 2022) and SLAK (Liu et al., 2023) demonstrate the importance of receptive field enlargement by directly adopting ultra-large kernels through structural re-parameterization and sparsification, yet their physical large-kernel designs still suffer from hardware compatibility and computational efficiency issues. Shift-ConvNets (Li et al., 2025) decompose large kernels into small kernels combined with shift-wise operations, improving hardware friendliness, but rely on fixed shift strides and thus lack dynamic adaptability for multi-scale object modeling.

**Figure 3:**
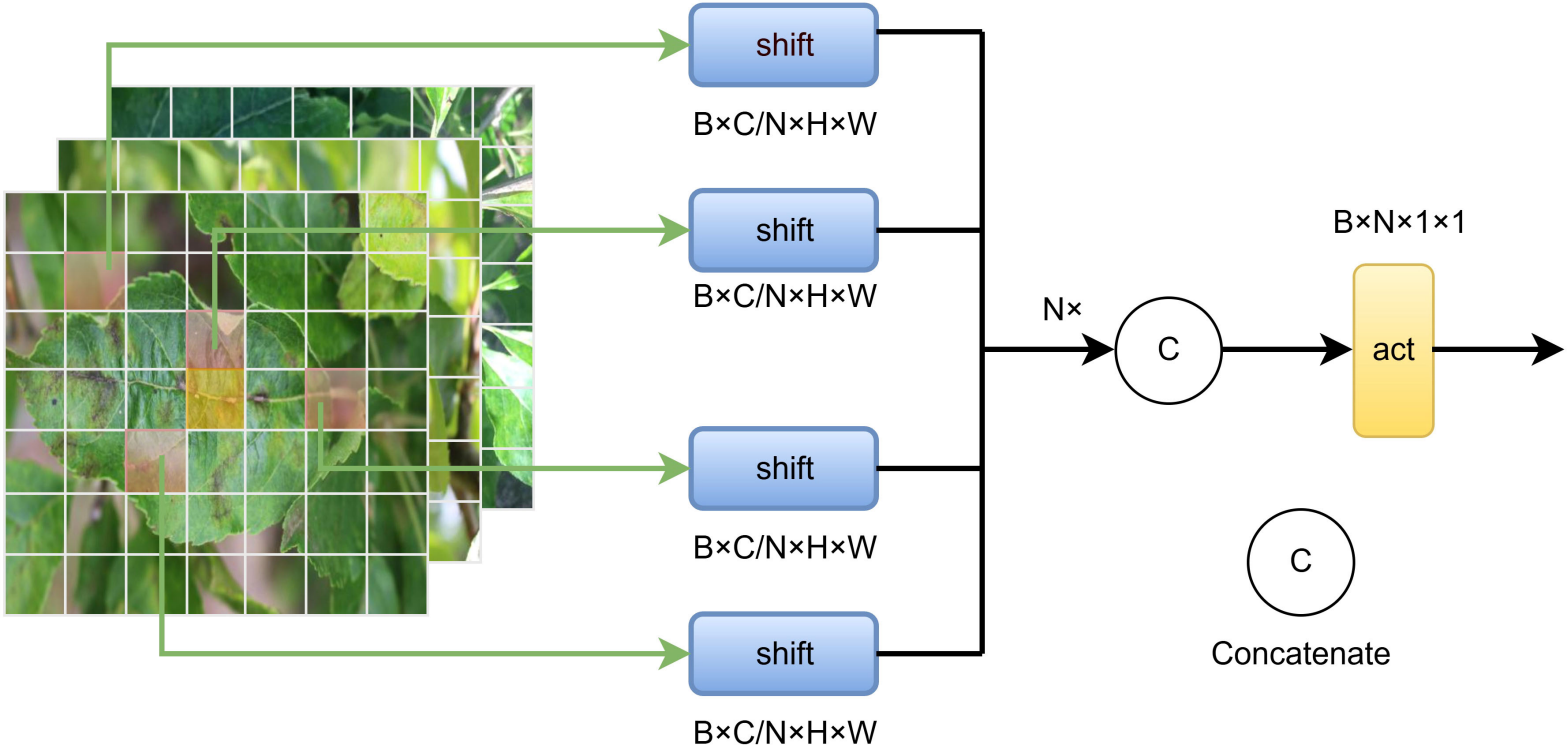
Dynamic channel shift module structure.

To overcome these limitations, we propose a DCS module, which introduces learnable dynamic shifts combined with cross-scale feature interaction. This design enables a larger effective receptive field while maintaining identical kernel sizes, thereby enhancing multi-scale representation capability under lightweight constraints. The overall architecture of DCS is illustrated in **Figure 3**.

Given an input feature map ${\text{ X}}_{\text{in}}\in {\text{R}}^{\text{B}\times {\text{C}}_{\text{in}}\times \text{H}\times \text{W}}$,the module produces an output feature map $\text{Y}\in {\text{R }}^{\text{B}\times {\text{C}}_{\text{out}}\times \text{H}\times \text{W}}$.The output is generated through the following three steps.

Standard convolution and local feature extraction.A standard convolution is first applied to extract local features, followed by batch normalization and a nonlinear activation (Equation 11):

(11) $$\tilde{X}=\delta \left(BN\left(W_{c}*X_{in}\;\right)\right)$$

Channel-wise dynamic shift modeling.The feature map $\tilde{X}$ is evenly divided into G channel groups, each containing ${\text{C}}_{\text{g}}=\frac{{\text{C}}_{\text{out}}}{\text{G}}$ channels. For the g -th group, an independent spatial shift is applied (Equation 12):

(12) $${\tilde{X}}_{g}=Roll\left(X_{g},\left(\text{Δ}h_{g},\text{Δ}w_{g}\right)\right),g=1,2,\ldots ,G$$

where ${\text{X}}_{\text{g}}$ denotes the feature subset of the g-th group, with ${\text{X}}_{\text{g}}\in {\text{R}}^{\text{B}\times {\text{C}}_{\text{g}}\times \text{H}\times \text{W}}$; Roll(·) represents a circular shift operation, and ${\text{Δh}}_{\text{g}}$ and ${\text{Δw}}_{\text{g}}$ are learnable shift offsets of the g-th group along the height and width dimensions,respectively.

Feature reassembly and fusion.All shifted groups are concatenated along the channel dimension to form the output feature map (Equation 13):

(13) $$X_{out}=Concat\left({\tilde{X}}_{1},{\tilde{X}}_{2},\ldots ,{\tilde{X}}_{G}\right)$$

This process allows DCS to approximate multiple convolution kernels with dynamic receptive fields, thereby strengthening feature representation.

**Figure 4:**
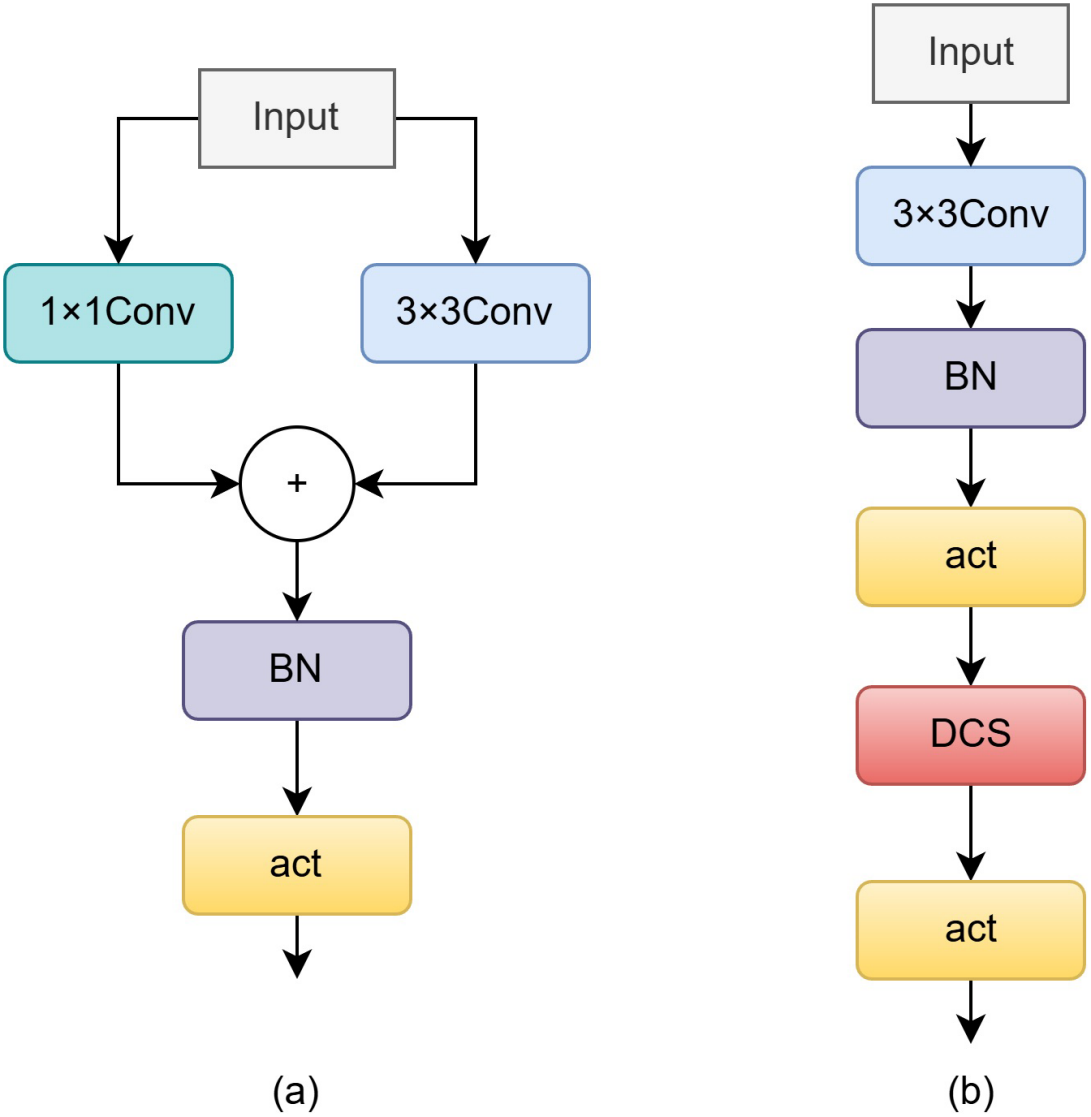
Structural comparison of different architectures. **(a)** RepVGG-style multi-branch structure. **(b)** Proposed single-path DCS structure for intuitive illustration.

In the original RT-DETRv2 design, a multi-branch structure is adopted during feature fusion (**Figure 4a**) to enhance gradient diversity and representation capacity. In contrast, we employ a single-path structure (**Figure 4b**) for three main reasons: (1) to simplify the training process by avoiding the extra computation and memory overhead introduced by multiple branches; (2) to improve deployment convenience without requiring complex parameter merging; and (3) as validated in **Table 1**. Impact of different feature fusion structures on detection performance., the contribution of multi-branch structures to detection accuracy becomes marginal after integrating BCG and DCS.

**Table 1:** Impact of different feature fusion structures on detection performance.

Variant	mAP	Params	Flops
multi-branch	0.644	20.1	40.0
​sequential	0.6434	19.9	39.5

### 2.5 Loss

In object detection, especially Transformer-based end-to-end approaches, loss function design is as important as model architecture innovation. A core challenge lies in efficiently guiding sparse queries to match ground truth objects and optimizing them.

Existing methods have explored different directions but remain limited. Focal Loss (FL) (Lin et al., 2017) addresses class imbalance via a modulation factor, but its objective uses static binary labels and does not link classification confidence with localization quality. Varifocal Loss (VFL) (Zhang et al., 2021) softens target values to IoU but produces shallow gradients in low-IoU regions, resulting in insufficient optimization for many low-quality matches. Matchability-Aware Loss (MAL) (Huang et al., 2025) introduces IoU-based matchability modeling to distinguish high- and low-quality matches, improving training stability and optimization of low-quality samples.

It should be noted that the MAL paper contains formula ambiguities that could lead to negative losses if implemented directly. However, its official GitHub implementation already incorporates smoothing mechanisms to improve training on low-IoU samples.

Building upon this insight, we adopt and reformulate MAL into a smoother and more stable variant, termed Smooth Matching Loss (SML). Rather than introducing a completely new loss function, SML can be regarded as an implementation-oriented refinement of MAL, with improved numerical stability and clearer formulation. The core design of SML is as follows:

Positive sample loss.Introduces both matchability constraint and negative-sample focusing in positive samples, unifying positive and negative expressions and ensuring gradient continuity (Equation 14):

(14) $$L_{pos}=-q^{\gamma }log\left(p\right)-\left(1-q^{\gamma }\right)log\left(1-p\right)$$

where p denotes predicted confidence, q denotes IoU between predicted and ground truth boxes, and γ is a modulation factor. As q→1, it degenerates to standard BCE; as q→0, it smoothly transitions toward the negative sample form.

Negative sample loss. Retains the negative-sample focusing mechanism from Focal Loss (Equation 15):

(15) $$L_{neg}\;=\;-p^{\gamma \;}log\left(1\;-\;p\right)$$

SML unifies positive and negative sample expressions while preserving IoU-based matchability, ensuring gradient continuity and improving low-quality match optimization;Introduces constraints on high-confidence mismatches (false positives), reducing overconfident predictions.

The overall loss is defined as (Equation 16):

(16) $$L\;=\;L_{pos}+\;L_{neg}$$

## 3 Results

### 3.1 Dataset

**Figure 5:**
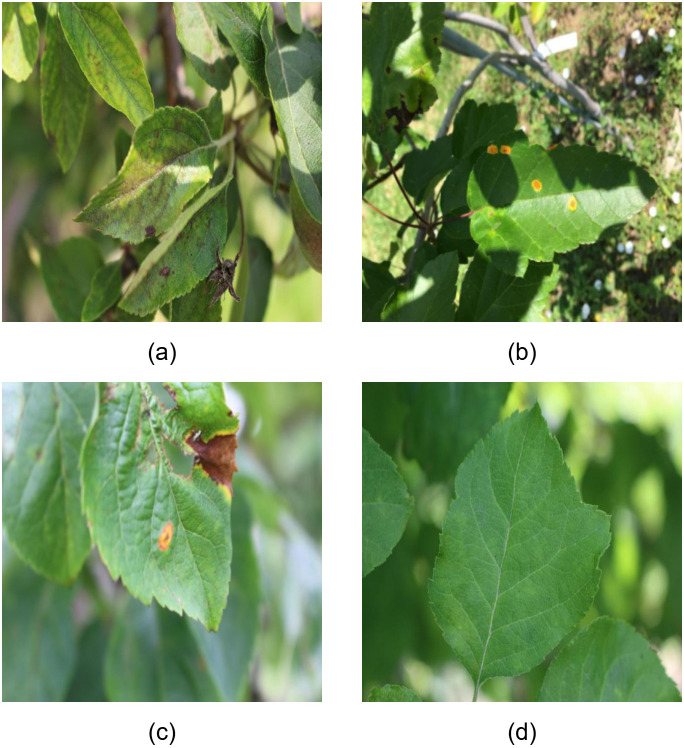
Sample images from the plant-disease dataset. **(a-c)** show three types of plant diseases, while **(d)** shows a healthy plant.

Experiments in this study are conducted on the publicly available Plant-Disease (Roboflow, [[NoYear]]a) dataset released on the RoboFlow platform. The dataset consists of four categories, including one class of healthy leaves and three classes corresponding to different plant disease symptoms. Representative samples of the four categories are shown in **Figure 5**.

The Plant-Disease dataset contains a total of 1782 annotated images, which are divided into training, validation, and test sets with 1422, 324, and 36 images, respectively. All images in the dataset have a fixed original resolution of 512 × 512 pixels, and no additional resizing is applied.

### 3.2 Evaluation metrics

To comprehensively evaluate the performance of the proposed method for plant disease detection, evaluation metrics are selected from two aspects: detection accuracy and computational efficiency, including mean Average Precision (mAP), mean Average Recall (mAR), Floating Point Operations (FLOPs), and the number of model parameters (Params). Specifically, mAP and mAR are used to assess detection performance, while FLOPs and Params reflect computational complexity and deployment cost.

Precision and Recall are defined as (Equations 17, 18):

(17) $$\;Precision=\frac{TP}{TP+FP}$$

(18) $$\;Recall=\frac{TP}{TP+FN}$$

where TP denotes true positives, representing correctly detected targets; FP denotes false positives, indicating background or incorrect regions mistakenly detected as targets; and FN denotes false negatives, referring to existing targets that are not detected by the model.

mAP evaluates the overall detection performance by averaging the area under the precision–recall curve for each category (Equation 19):

(19) $$mAP\;=\;\frac{\sum_{i=1}^{n}\;\int_{0}^{1}P_{i}\left(R\right)dR\;}{n}$$

where n is the number of categories and ${\text{P}}_{\text{i}}\left(\text{R}\right)$ represents the precision of the i-th class at recall level R. In this study, COCO-style evaluation is adopted, and mAP is computed over multiple IoU thresholds.

mAR is defined as the average recall across all categories (Equation 20):

(20) $$mAR\;=\;\frac{\sum_{i=1}^{n}\;R\left(i\right)\;}{n}$$

Where R(i) denotes the recall of the i-th category under the given detection setting. This metric reflects the model’s overall capability to cover targets of different plant disease classes.

To evaluate computational efficiency, FLOPs and Params are employed as complexity metrics. For a standard convolutional layer, FLOPs and parameter count can be expressed as (Equations 21, 22):

(21) $$Flops=2HW\left(C_{in}K^{2}+1\right)C_{out}$$

(22) $$Params=\left(C_{in}K^{2}+1\right)C_{out}$$

where H and W denote the spatial height and width of the output feature map, ${\text{C}}_{\text{in}}$ and ${\text{C}}_{\text{out}}$ represent the numbers of input and output channels, respectively, and K is the kernel size. The additional term accounts for bias parameters.

### 3.3 Implementation details

The proposed model is implemented based on PyTorch. The software and hardware environments are summarized in **Table 2**. Software and hardware configuration for experiments. During training, the AdamW optimizer is used for 120 epochs with a batch size of 16. The learning rate follows a cosine annealing schedule after warm-up, as detailed in **Table 3**. Training hyperparameter settings.

**Table 2:** Software and hardware configuration for experiments.

Name	Version
System	ubuntu22.04
Python	3.10
CUDA	11.8
CPU	15 vCPU Intel(R) Xeon(R) Platinum 8358P CPU @ 2.60GHz
GPU	RTX 3090(24GB) * 1

**Table 3:** Training hyperparameter settings.

Type	T_Max	LR_Min	Warmup_duration	LR_Max
LinearWarmup	–	–	500	0.0004
CosineAnnealingLR	150	0.00005	–	–

### 3.4 Ablation studies

To verify the effectiveness and synergy of each improvement module, ablation studies are conducted using RT-DETRv2 as the baseline under the same experimental settings and hyperparameters. The results are presented in **Table 4**. Ablation study results of different modules on the Plant-Disease dataset.

**Table 4:** Ablation study results of different modules on the plant-disease dataset.

Model	BCG	DCS	SML	mAP50(%)	mAP75(%)	mAP50:95(%)	mAR(%)	Flops(G)	Params(M)
Baseline	–	–	–	88.5	75.7	62.7	75.1	40.0	20.1
1	✔	–	–	90.5	77.9	63.3	73.8	40.1	20.2
2	–	✔	–	90.2	78.5	64.3	74.8	39.5	19.9
3	–	–	✔	89.6	77.3	63.7	73.6	40.0	20.1
4	✔	✔	–	92.7	78.7	66.6	75.9	39.5	20.0
Ours	✔	✔	✔	93.6	81.2	67.2	75.4	39.5	20.0

Notably, combining the BCG and DCS modules brings a 4.2% improvement in mAP, indicating that the modules are functionally complementary: BCG enhances feature representation, while DCS mitigates the extra computation overhead. The full model, integrating BCG, DCS, and SML, achieves the best performance on the Plant-Disease dataset: mAP50 reaches 93.6% (+5.1%), mAP75 81.2% (+5.5%), and overall mAP 67.2% (+4.5%). Meanwhile, mAR slightly improves from 0.751 to 0.754, while FLOPs (39.5G) and Params (20.0M) remain comparable to the baseline (40.0G and 20.1M). These results demonstrate that the proposed improvements enhance detection accuracy without significantly increasing computational costs, achieving a better accuracy-efficiency trade-off.

### 3.5 Comparison experiments

To evaluate the practical performance of the proposed model, comparative experiments are conducted on the public Plant-Disease dataset. All models are trained and evaluated under the same experimental settings to ensure a fair comparison. The proposed method is systematically compared with mainstream one-stage detectors and representative DETR-based models, as summarized in **Table 5**. Performance comparison with state-of-the-art object detection models on the Plant-Disease dataset.

**Table 5:** Performance comparison with state-of-the-art object detection models on the plant-disease dataset.

Model	Params(M)	Flops(G)	mAP50(%)	mAP(%)
YOLOv8m	25.9	50.5	88.8	63.3
YOLOv9m	20.2	49.2	91.8	65.5
YOLOv10m	16.5	37.8	87.1	61.3
YOLOv11m	20.1	43.5	88.8	63.0
YOLOv12m	20.1	43.2	87.9	60.1
Deformable DETR	39.9	110.7	90.7	55.9
DINO DETR	46.8	178.6	90.0	61.3
RTDETRv2	20.1	40.0	88.5	62.7
DEIM	19.5	38.1	92.6	65.8
ours	20.0	39.5	93.6	67.2

The compared one-stage detectors include YOLOv8m, YOLOv9m, YOLOv10m, YOLOv11m (Khanam and Hussain, 2024), and YOLOv12m (Tian et al., 2025). In addition, representative end-to-end detectors, including Deformable DETR, DINO, RT-DETR, and DEIM, are also considered for comparison.

In terms of model complexity, the proposed method contains 20.0 M parameters and requires 39.5 G FLOPs, which is comparable to RT-DETR and DEIM, and only slightly higher than YOLOv10m. This indicates that the proposed model maintains favorable lightweight characteristics and deployment feasibility.

Regarding detection accuracy, the proposed model achieves the best performance under different IoU thresholds. Specifically, it reaches 93.6% mAP50 and 67.2% mAP, outperforming the strongest baseline YOLOv9m by 1.8% and 1.7%, respectively. Moreover, compared with DETR-based models that require substantially higher computational costs, the proposed method achieves a more balanced trade-off between accuracy and efficiency.

Overall, these results demonstrate that the proposed model significantly improves detection accuracy without introducing excessive computational overhead, highlighting its effectiveness for real-time plant disease detection in practical agricultural scenarios.

### 3.6 Generalization experiment

To further evaluate the robustness and generalization capability of the proposed method in plant disease detection, cross-dataset experiments are conducted on two independent public datasets. These datasets contain images captured under diverse field environments and growth stages, enabling evaluation beyond the original dataset.

The first is the Detecting Rice Crop Diseases dataset (Roboflow, [[NoYear]]b), which includes healthy samples and seven types of rice diseases collected under diverse environmental conditions, covering growth stages from leaf development to grain formation. The original image resolution is 416×416, and the dataset is divided into 2871 training images, 460 validation images, and 421 testing images.

The second is the disease detection dataset (Roboflow, [[NoYear]]c), which consists of healthy samples and two types of lettuce diseases collected under various environmental conditions and growth stages. The images have a resolution of 640×640, with 1338 images for training, 227 for validation, and 106 for testing.

These datasets represent detection scenarios ranging from multi-disease, multi-category crops to single-crop disease detection, providing a comprehensive evaluation of model generalization under diverse conditions.

The ablation results on the two additional datasets are presented in **Table 6**. Ablation study on the Detecting Rice Crop Diseases dataset. and **Table 7**, corresponding to the Detecting Rice Crop Diseases dataset and the disease detection dataset, respectively.

**Table 6:** Ablation study on the detecting rice crop diseases dataset.

Model	BCG	DCS	SML	mAP50(%)	mAP75(%)	mAP50:95(%)	mAR(%)
Baseline	–	–	–	49.4	24.0	26.9	24.8
1	✔	–	–	50.0	25.7	28.0	26.2
2	–	✔	–	47.9	25.6	27.6	26.2
3	–	–	✔	46.9	25.6	27.3	25.8
Ours	✔	✔	✔	52.0	26.5	28.9	25.0

**Table 7:** Ablation study on the disease detection dataset.

Model	BCG	DCS	SML	mAP50(%)	mAP75(%)	mAP50:95(%)	mAR(%)
Baseline	–	–	–	56.8	37.7	37.1	22.7
1	✔	–	–	62.0	39.5	39.6	27.3
2	–	✔	–	64.2	40.2	40.2	26.9
3	–	–	✔	63.0	39.4	40.3	27.4
Ours	✔	✔	✔	68.3	39.2	41.8	27.4

On the Detecting Rice Crop Diseases dataset, the proposed method achieves overall improvements compared with the baseline model. The final model reaches 52.0% mAP50, improving the baseline result of 49.4% by 2.6 percentage points, while mAP50:95 increases from 26.9% to 28.9%. Although the improvements of individual modules vary across metrics, the combined model consistently achieves the best overall performance. These results indicate that the proposed modules remain effective under different crop types and disease characteristics.

On the disease detection dataset, all proposed modules contribute to performance improvement compared with the baseline model. When the modules are introduced individually, the model achieves noticeable gains in detection performance. Specifically, the BCG module increases mAP50 from 56.8% to 62.0%, while the DCS and SML modules improve it to 64.2% and 63.0%, respectively. When all modules are combined, the final model achieves 68.3% mAP50 and 41.8% mAP50:95, representing improvements of 11.5% and 4.7% over the baseline. These results demonstrate that the proposed modules effectively enhance feature representation and detection performance on this dataset.

Overall, the results on both datasets demonstrate that the proposed modules contribute to improved detection performance and maintain reasonable generalization capability across different plant disease datasets.

### 3.7 Visual analytics

**Figure 6:**
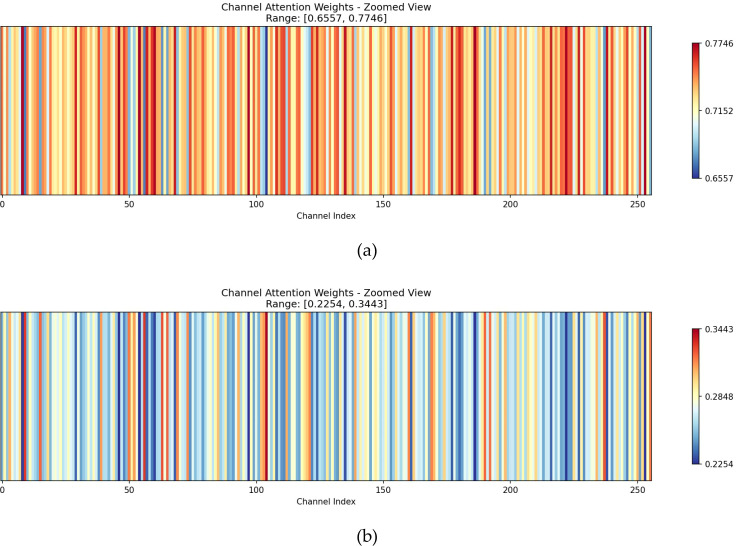
Visualization of channel attention weights. The heatmap display range is normalized according to the minimum and maximum channel weight values. **(a)** Weights for high-level features. **(b)** Weights for low-level features.

Bidirectional Cross Gate.To further investigate the working mechanism of the BCG module during feature fusion, this section visualizes and analyzes the bidirectional channel attention weights generated by the module. **Figure 6** illustrates the channel attention weight distributions corresponding to high-level and low-level features (shown with enlarged views).

As shown in **Figures 6a, the** channel attention weights associated with high-level features are mainly distributed within the relatively high range of [0.6557,0.7746], with a mean value significantly greater than 0.5. This distribution indicates that, during feature fusion, the model tends to assign higher importance to channels originating from high-level features, which is consistent with the prior knowledge that high-level features contain richer semantic information and should dominate the fusion process.

In contrast, **Figure 6b** shows that the channel attention weights for low-level features are primarily concentrated in a lower range of [0.2254,0.3443], forming an approximately complementary relationship with the high-level weights. This observation aligns with the Softmax normalization constraint in Equation (8), demonstrating that the attention mechanism can adaptively allocate importance across feature levels along the channel dimension, thereby enabling effective information selection.

Moreover, during the bidirectional cross-injection process, the relatively low weights assigned to low-level features indicate that their contribution to high-level feature enhancement is controlled and complementary. This design allows spatial detail information from low-level features to be incorporated without excessively disturbing the core semantic representation of high-level features. Overall, the observed weight distributions validate the effectiveness of the BCG design, showing that the model can automatically learn cognitively consistent importance allocation between semantic and detail features, leading to more stable and effective cross-level feature fusion.

Dynamic Channel Shift.To systematically evaluate the effectiveness of the proposed DCS structure, we conduct an Effective Receptive Field (ERF) analysis, with the RepVGG block selected as the baseline architecture.

**Figure 7:**
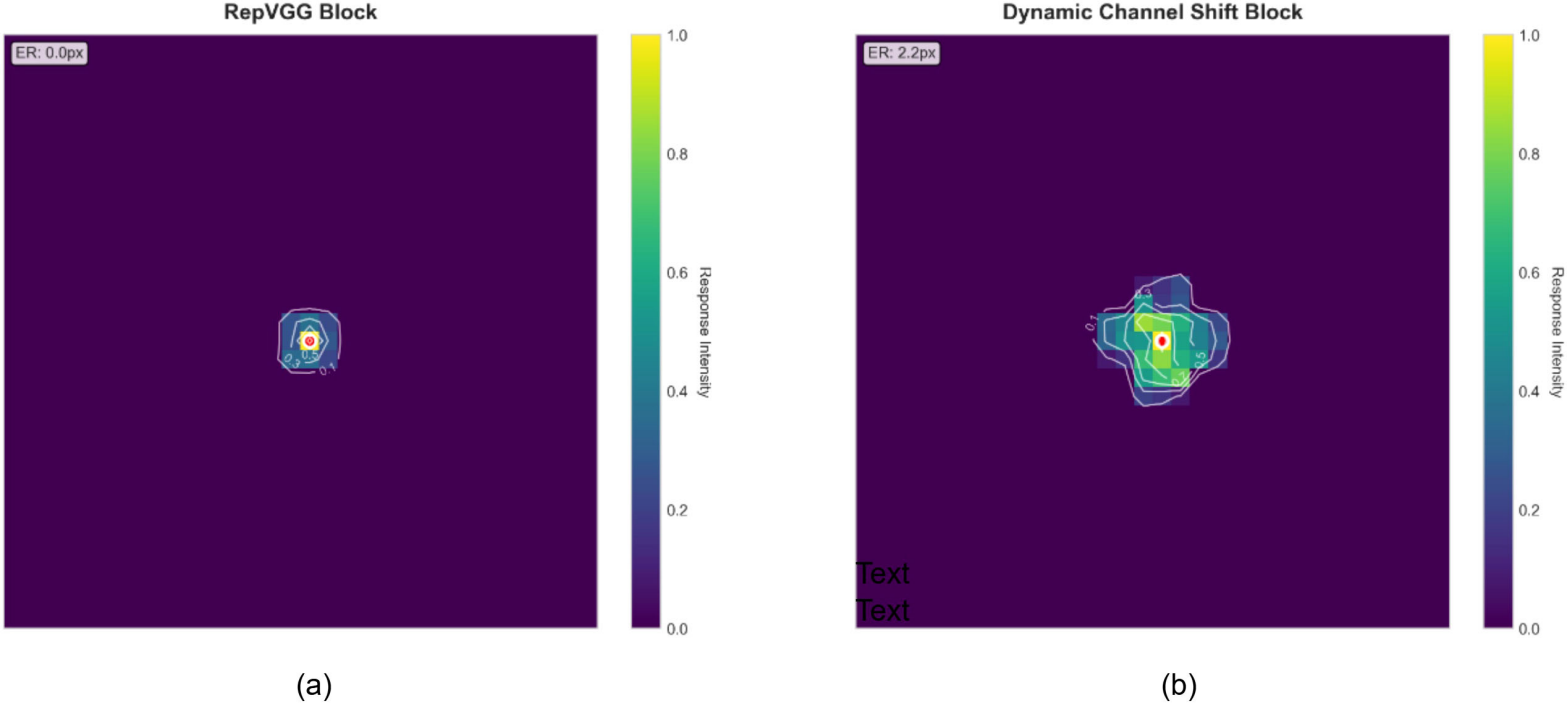
Comparison of effective receptive fields between the RepVGG block and the DCS block. **(a)** Heatmap of the effective receptive field for the RepVGG block. **(b)** Heatmap of the effective receptive field for the DCS block.

**Figure 7** presents a comparison of the spatial response distributions of the RepVGG block and the DCS block, enabling both qualitative and quantitative assessment of their receptive field characteristics.

As shown in **Figures 7a**, the RepVGG Block exhibits a highly concentrated response pattern, with an effective receptive field radius (ER) of only 0.0 pixels based on the impulse response definition. In the corresponding heatmap, the high-response region (yellow core) is strictly confined to the center, and the peripheral responses (ranging from green to blue) decay rapidly, indicating that this structure has a highly localized perception of the input space. This response characteristic reflects the nature of standard convolutional structures, which focus on local receptive fields. Although it can precisely model features in the central region, it inherently has limitations in capturing long-range contextual dependencies.

In contrast, the DCS Block, as shown in **Figures 7b**, demonstrates a significantly expanded receptive field, with its effective receptive field radius increasing to 2.2 pixels. Its response distribution is more dispersed, with a markedly enlarged high-response area and a more gradual decay from the center to the periphery. This phenomenon is mainly attributed to the channel shift mechanism introduced by DCS, which, through multiple groups of spatial shift operations, effectively simulates the effect of large-kernel convolutions at the feature level without explicitly introducing large-kernel parameters, thereby enhancing the model’s ability to integrate contextual information.

**Figure 8:**
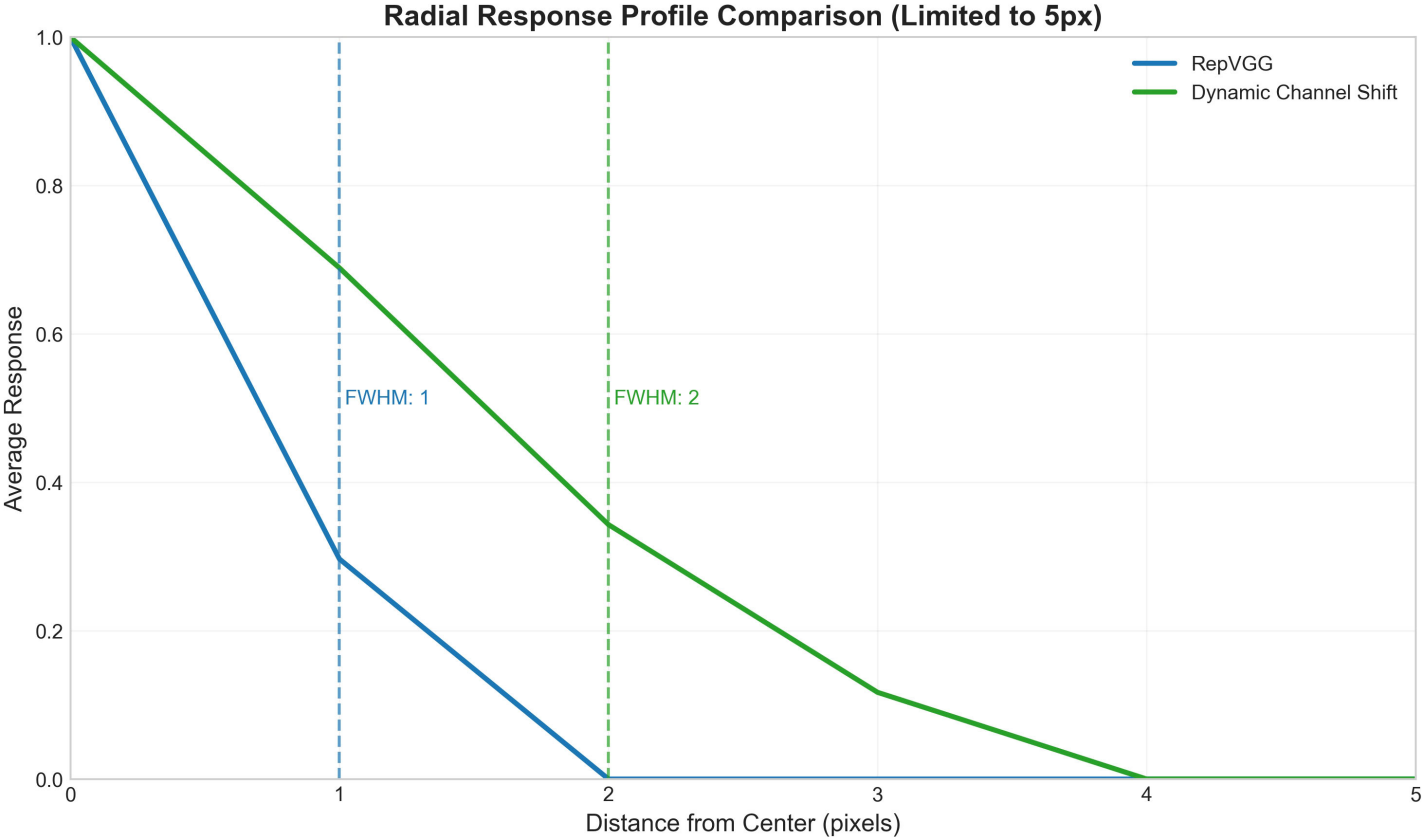
Comparison of radial average response profiles between the RepVGG Block and the DCS Block.

To further analyze the spatial response decay characteristics of the two structures, the radial average response profiles are plotted in **Figure 8**. To emphasize the core differences, the horizontal axis in **Figure 8** (pixel distance from the center) is limited to within 5 pixels. Overall, both curves start with high response values at 0 pixels from the center and gradually decay with increasing distance. However, the decay rates of the two structures differ significantly: the response of the RepVGG Block (blue curve) drops rapidly, with a full width at half maximum (FWHM) of only 1 pixel, whereas the decay of the DCS Block (green curve) is more gradual, with its FWHM extending to 2 pixels. This indicates that the DCS structure maintains significant non-zero responses even at farther spatial positions, quantitatively validating its advantage in expanding the effective receptive field.

**Figure 9:**
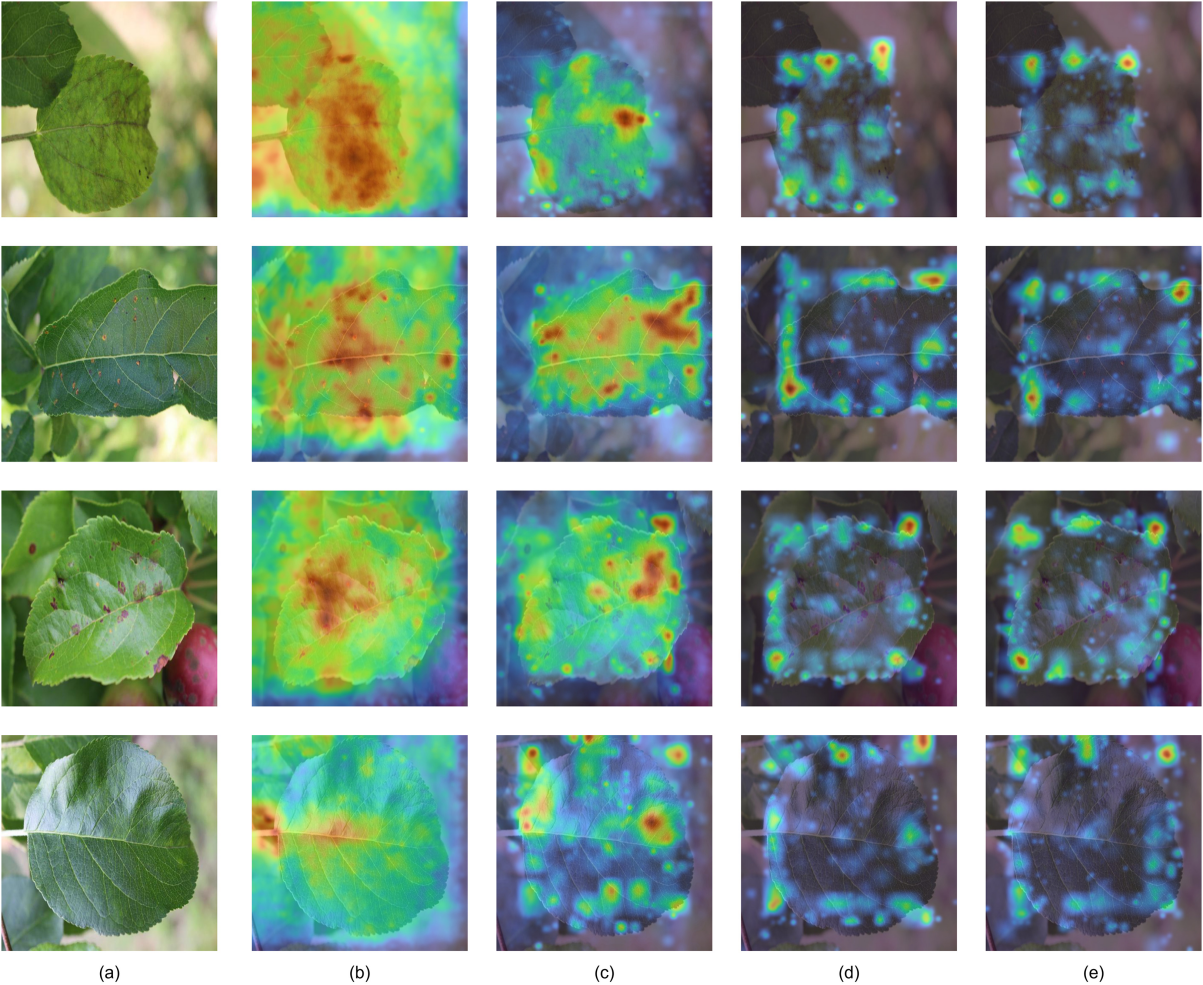
Visualization of decoder attention heatmaps. **(a)** Input image. **(b)** All-query attention of the improved model. **(c)** All-query attention of the baseline model. **(d)** Detection-query attention of the improved model. **(e)** Detection-query attention of the baseline model.

Attention Heatmap. To further investigate the impact of the proposed structural improvements on feature representation and detection behavior, we visualize and compare the decoder attention distributions of the baseline and improved models, as shown in **Figure 9**. To avoid ambiguity in interpretation, the decoder attention visualizations are divided into two categories.

1. All-query attention aggregates the cross-attention of all decoder queries and reflects the global attention behavior during the feature fusion stage.
2. Detection-query attention considers only queries associated with final predictions and reflects target-level localization and discrimination behavior.

These two types of attention correspond to the spatial distribution characteristics and the discriminative capability of the model, respectively.

For the all-query attention heatmaps, the visualization reflects how the model distributes attention across the global feature space rather than focusing on specific targets. In healthy plant samples, the improved model exhibits a more uniform and smoother attention distribution with fewer localized high-response regions. This indicates that the introduced mechanisms reduce unnecessary responses to non-discriminative regions, such as leaf textures and background noise, resulting in a more stable global representation. In diseased samples, compared with the baseline model, the improved model shows a more distributed attention pattern. This behavior suggests that the model no longer overly relies on isolated local lesion cues but instead captures disease-related information within a broader spatial context, which is beneficial for representing complex and irregular lesion patterns.

In contrast, the detection-query attention heatmaps directly reflect the model’s behavior during the detection stage. For diseased samples, the improved model produces attention that is more consistently aligned with lesion regions, while the baseline model is more easily affected by background interference. For healthy samples, the improved model avoids generating strong localized responses, indicating effective suppression of false activations and reducing the risk of misclassification. These observations suggest that the proposed improvements enhance both localization reliability and robustness against background noise.

**Figure 10:**
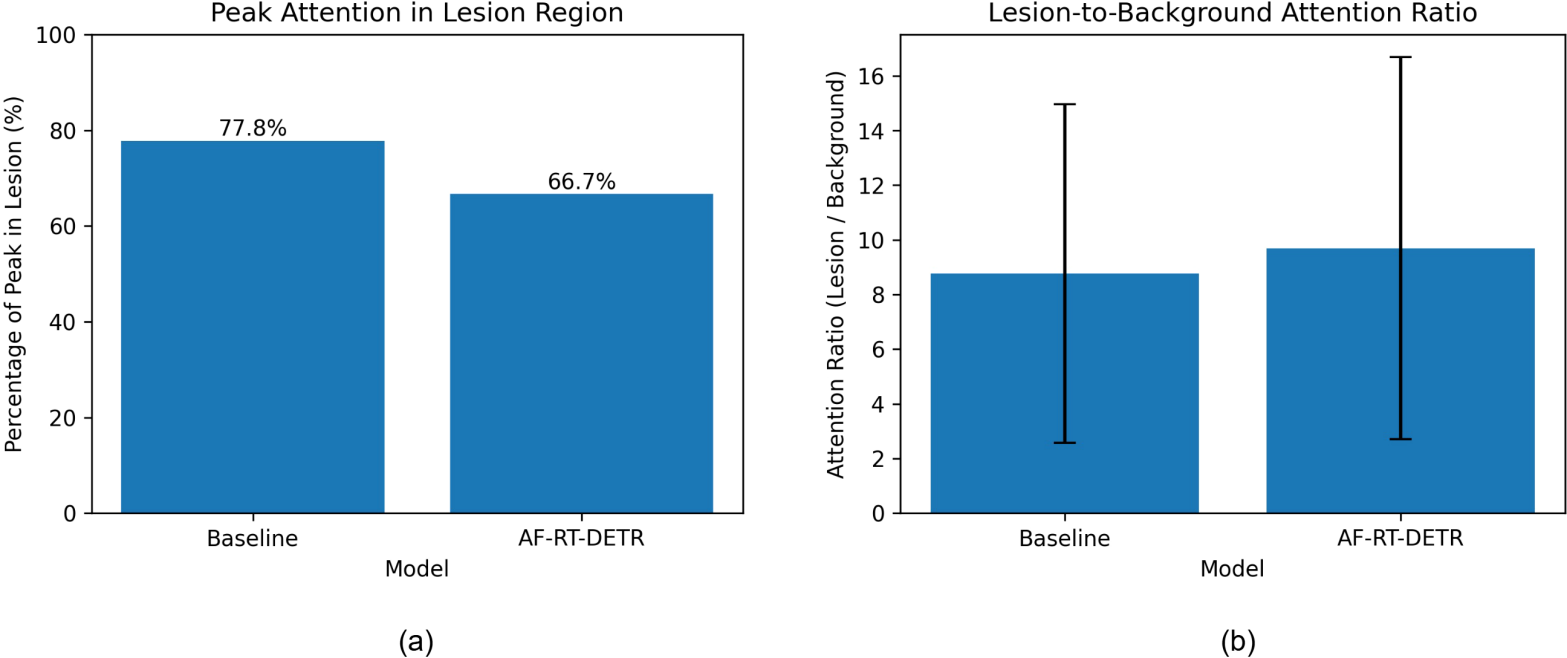
Comparison of attention distribution in lesion regions. **(a)** Percentage of peak attention located in lesion regions. **(b)** Lesion-to-background attention ratio of the baseline model and AF-RT-DETR.

To further support these observations, statistical analysis is conducted over the entire test set, as shown in **Figure 10**. Two complementary metrics are adopted to characterize different aspects of attention behavior.

1. Attention Peak Accuracy (APA) measures whether the maximum attention response falls within the ground-truth lesion region, reflecting the model’s tendency to form highly concentrated peak responses in target areas.
2. Lesion-to-Background Attention Ratio (LBAR) is defined as the ratio between the average attention within lesion regions and that in background regions, reflecting the overall discriminative capability between target and non-target areas.

The results show that the baseline model achieves a higher APA, indicating that its attention is more likely to form highly concentrated peaks within lesion regions. In contrast, the improved model obtains a higher LBAR, suggesting better overall separation between lesion and background regions across the spatial domain.

It is important to note that these two metrics reflect different characteristics of attention distribution and imply a trade-off. A higher APA is typically associated with more concentrated attention, which favors peak localization but may increase reliance on limited local features. A higher LBAR, on the other hand, indicates that the model enhances the overall response contrast between lesion and background regions, even if the absolute peak is not always located within the lesion area.

This relationship is consistent with the observations in **Figure 9**. The baseline model exhibits more localized and concentrated responses, which contributes to its higher APA. In contrast, the improved model produces more spatially coherent and context-aware attention distributions, leading to improved overall contrast and thus higher LBAR. Therefore, the improved model shifts from a peak-focused attention pattern to a more globally discriminative representation.

Overall, these results demonstrate that the proposed structural improvements maintain reasonable spatial focus while enhancing the model’s ability to distinguish lesion regions from the background, leading to more stable and reliable detection behavior.

## 5 Discussion

This study addresses the practical challenges of plant disease detection in complex field environments by introducing targeted improvements to the RT-DETRv2 framework without significantly increasing model parameters or computational cost. Experimental results demonstrate that the proposed modifications enhance detection accuracy and robustness at multiple levels, with improvements reflected not only in quantitative metrics but also in more interpretable feature representations and model behavior.

The introduced bidirectional cross-gating mechanism enables adaptive importance allocation among multi-scale feature representations, facilitating effective interaction between high-level semantic information and low-level spatial details. This property is particularly beneficial in real-world agricultural scenarios characterized by leaf occlusion, illumination variation, and large-scale diversity of disease symptoms, as it helps maintain stable feature representations under complex backgrounds. In addition, the DSC structure expands the effective receptive field without relying on physically large convolution kernels, thereby improving contextual modeling capability while preserving hardware efficiency and deployment flexibility. Such a design aligns well with the real-time and resource-constrained requirements of agricultural applications.

At the loss function level, the proposed smooth matching loss unifies the optimization of positive and negative samples through a continuous formulation across different matching quality regions. This design improves training stability in scenarios dominated by low-quality matches and reduces the adverse impact of mismatched samples on model convergence and confidence estimation, contributing to enhanced robustness under complex disease distributions.

Although the proposed method demonstrates strong performance and generalization across multiple public datasets, there remains room for improvement in extremely dense small-object or heavily occluded scenarios. Future work may explore more refined multi-scale supervision strategies or incorporate temporal information to further enhance applicability in real-world agricultural production environments.

## 6 Conclusion

This paper proposes a plant disease detection model based on the RT-DETRv2 architecture, aiming to improve detection performance while maintaining comparable parameter size and computational complexity.

At the feature extraction stage, a bidirectional cross-gating mechanism is introduced to enable effective information interaction among multi-scale feature maps, thereby alleviating channel redundancy.

At the feature fusion stage, the original RepVGG module is replaced with a DSC module, which significantly expands the effective receptive field while maintaining model lightweightness and enhances the modeling of contextual information.

In addition, an improved multi-scale alignment loss is designed to strengthen supervision on low-quality feature maps, further improving the robustness of the model in complex scenarios.

Experimental results demonstrate that the proposed method effectively enhances detection accuracy and generalization performance for plant disease detection while preserving the lightweight characteristics of the model, validating the effectiveness of the overall improvement strategy.

## Data Availability

The original contributions presented in the study are included in the article/supplementary material. Further inquiries can be directed to the corresponding author.
